# Recovery and long term functional outcome in people with critical illness polyneuropathy and myopathy: a scoping review

**DOI:** 10.1186/s12883-022-02570-z

**Published:** 2022-02-11

**Authors:** Domenico Intiso, Antonello Marco Centra, Michelangelo Bartolo, Maria Teresa Gatta, Michele Gravina, Filomena Di Rienzo

**Affiliations:** 1 Unit of Neuro-Rehabilitation and Rehabilitation Medicine, IRCCS “Casa Sollievo della Sofferenza”, Viale dei Cappuccini, 71013 San Giovanni Rotondo (FG), Italy; 2 Department of Rehabilitation, Neurorehabilitation Unit, Habilita Care & Research, Zingonia (Bergamo), Italy

**Keywords:** Critical illness polyneuropathy, ICU acquired weakness, Functional outcome, Rehabilitation

## Abstract

**Background:**

Intensive care unit acquired weakness (ICUAW), embraces an array of disorders labeled “critical illness polyneuropathy” (CIP), “critical illness myopathy” (CIM) or “critical illness polyneuromyopathy” (CIPNM). Several studies have addressed the various characteristics of ICUAW, but the recovery is still unclear.

**Objective:**

The present review investigated the recovery and the long-term functional outcome of subjects with ICUAW, whether the types of ICUAW have different outcomes and whether there is any supporting evidence.

**Methods:**

Literature search was performed from MEDLINE/PubMed, CINAHL, EMBASE, PeDro, Web of Science and Scopus. Inclusion criteria were: i) sample size including five or more subjects; ii) subjects who suffered from ICUAW and/or CIP, CIM and CIP/CIM; iii) ICUAW ascertained by EMG. Follow-ups longer than one year were defined as long-term.

**Results:**

Twenty-nine studies met the inclusion criteria. In total, 788 subjects with ICUAW were enrolled: 159 (20.1%) died and 588 (74.6%) were followed. Of all the included patients, 613 (77.7%) had CIP, 82 (10.4%) CIM and 56 (7.1%) CIP/CIM. Overall, 70.3% of the subjects with ICUAW fully recovered. Seven (24.1%) studies had a follow-up longer than 1 year (range 2–8) with 173 (21.9%) subjects enrolled globally and 108 followed. Of these subjects, 88.8% gained full recovery. Most of the studies did not use proper functional scales and only 4 and 3 studies employed the Barthel scale and the Functional Independence Measure (FIM) scale. Differentiation between the types of ICUAW was performed in 7 studies, but only 3 studies reported that subjects with CIM had a better prognosis and earlier recovery than subjects with CIP/CIM.

**Conclusions:**

Subjects with ICUAW could achieve good recovery and could improve at follow-up. However, the quality of the published studies due to short follow-ups and the paucity of defined outcome measures require confirms.

**Supplementary Information:**

The online version contains supplementary material available at 10.1186/s12883-022-02570-z.

## Introduction

A number of studies have been published about the muscle weakness that may affect intensive care unit (ICU) survivors. This disorder, which in the intensive care literature is better known as ICU acquired weakness (ICUAW), embraces a spectrum of clinical conditions. All of these conditions show variable levels of muscle strength impairment, from weakness to paralysis, which involve, bilaterally, the upper and lower limbs of ICU subjects. Although the clinical pictures are generally indistinguishable, ICUAW encompasses different pathological forms that damage the muscular and the peripheral nervous system. Different types of this pathology have been described and labeled according to the histological aspects and the electrophysiological findings and depending on the predominant structure involved. In particular, the definitions include: i) critical illness polyneuropathy (CIP), if the peripheral nervous system is affected; ii) critical illness myopathy (CIM), if the muscles are involved, and iii) critical illness polyneuropathy and myopathy (CIP/CIM), critical illness neuromyopathy [1], and polyneuromyopathy (CIPNM) [2, 3], if the pathological process affects both muscles and nerves. Although ICU specialists prefer to use the term ICUAW, the definition “critical illness polyneuromyopathy” (CIPNM) is widely diffuse, but for the purposes of the present study, ICUAW term was used. After the first description by Bolton et al. at the beginning of the’80 s [4], a number of studies have been published that have contributed to making remarkable advances in the understanding of the complex aspects of ICUAW, such as the electrophysiological [5] and histological features [6] as well as the pathogenic mechanisms. The occurrence of this disorder has been variously detected with a range from 45 to 80% [7–9]. A systematic review reported a median prevalence of 43% [10]. Furthermore, many risk factors have been suggested to favor the development of ICUAW, including sex (female), sepsis, ICU length of stay and multiple organ failure [11, 12]. Several therapeutic approaches and strategies have been proposed and evaluated for the management of ICUAW subjects, but pharmacological treatments have failed to prevent the occurrence and were ineffective in treating the disorder [13–15]. However, recent reviews and meta-analyses have demonstrated that early mobilization is associated with a lower likelihood of developing this clinical condition [16, 17]. Some reviews have addressed the various aspects of ICUAW, but they have not highlighted recovery, functional outcome and quality of life [18–20]. Despite the lack of treatments and the limitations of rehabilitative strategies, it was reported that 55–70% of subjects reached a full recovery after ICU discharge [21, 22], and that recovery depended on the type of ICUAW, as confirmed by the fact that CIM had an earlier and better functional outcome than CIP [22, 23]. In 2005, an extensive literature review of the neuromuscular sequelae of ICU subjects with critical illness reported that 68.8% of them made a complete recovery and regained the ability to walk independently. However, such review was limited by an insufficient number of patients with a long follow-up, leaving unsettled the question of whether deficits following ICUAW were persistent [21]. Since then, no extensive study investigating the functional outcome in these subjects have been carried out, and the issue remains unsolved. The aim of the present review was to investigate recovery, in particular the long-term functional outcome of subjects with ICUAW, whether the types of ICUAW have different outcomes and whether there is any supporting evidence.

## Materials and methods

A search of the studies having tested the functional outcome in subjects with ICUAW was conducted using MEDLINE/PubMed, the Cochrane Central Register of Controlled Trials, CINAHL, EMBASE, PeDro, Web of Science and Scopus databases. The search was restricted to English language reports published between January 1984 and April 2021. The search terms varied slightly from database to database but included “intensive care unit acquired weakness”, “ICUAW”, “critical illness polyneuropathy”, “CIP”, “critical illness myopathy”, “CIM”, “critical polyneuropathy and myopathy”, “CIPNM”, “CIP and CIM”; “CIP/CIM”, “acute tetraplegia”, “rehabilitation”, “functional outcome”, “recovery”, “physical therapy” and “mobilization”. Search limits included only adults. Conference abstracts/posters or articles that were not peer-reviewed were excluded. The literature search was conducted by three independent authors (MC, MB, FDR). Inclusion criteria were: i) sample size including five or more subjects; ii) subjects who suffered from ICUAW and/or the following types: CIP, CIM and CIP/CIM; iii) ICUAW ascertained by EMG; iv) studies with mixed samples that used the definition of ICUAW, but subjects with CIP, CIM or CIP/CIM were also considered; v) follow-up and outcome.

In order to avoid confounding results, studies were excluded if: i) they contained only the definition of the ICUAW, without any reference to the types of ICUAW or to CIP, CIM or CIP/CIM; ii) ICUAW was not ascertained by EMG; iii) reviews concerned ICUAW but the main aim of the study was not the outcome. Studies concerning children were excluded as well as subjects with ICUAW and COVID-19.

We defined as long-term follow-ups those follow-ups longer than 1 year. In this review, the pathological condition was counted as CIP if this acronym or definition was not specified in the studies analyzed. Due to the variability of the study designs, the functional measurements, the follow-ups, and the lack of data on the score of measurements, quantitative analysis was not possible. The research was conducted according to the Preferred Reporting Items for Systematic Reviews and the Meta-Analyses (PRISMA) diagram, depicting the selection of the articles searched for the study.

## Results

**Fig. 1:**
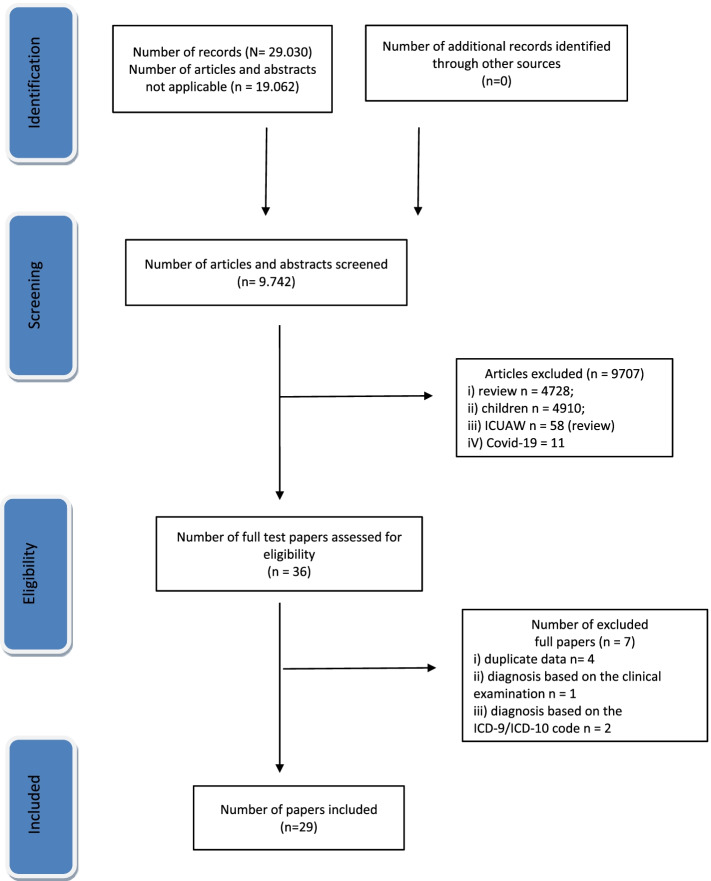
Preferred Reporting Items for Systematic Reviews and Meta-Analyses (PRISMA) diagram depicting the selection of articles for the study

The Prisma diagram of the studies’ selection is shown in Fig. 1. After studies were searched for and collected, 36 of them were considered eligible; of these, 29 [2, 6, 22–48] were included according to inclusion and exclusion criteria (Table 1). Seven studies [49–55] were excluded even if they included subjects with ICUAW (Appendix 1). In particular, 4 of these 7 studies were excluded because they contained a duplicate of the data already included in the 29 studies, where they were analyzed in a greater sample [48–51]; 1 study with a large sample, due to the diagnosis of ICUAW being based predominantly on the clinical examination [52]; 2 studies including ICU patients having been discharged with the ICD-9/ICD-10 code for CIP and CIM (53–55) and did not use functional scale scores [52, 53]. The included studies varied in aim, methodology design, sample size, case mix, inclusion/exclusion criteria, timing of the examination, follow-up and definition of recovery. Seventeen (58.6%) studies had a prospective design. Twenty-four (82.7%) studies concerned case series or small cohorts that had a mean sample size of 19.1 ± 7.7 patients and did not exceed the total number of 30 subjects. The other 5 studies [35, 41, 44, 45, 48] had samples greater than 30 patients (range 36–119) (Table 1). A total of 788 subjects with ICUAW were enrolled; of these 159 (20.1%) died and 588 (74.6%) were followed. All studies except 7 [6, 22, 23, 35, 41, 44, 46] did not perform the differentiation between the types of ICUAW and considered the disorder as a unique entity, labelling it as CIP or, more generically, as polyneuropathy or neuromuscular disorder. In particular, 613 (77.7%) patients had CIP, 82 (10.4%) CIM and 56 (7.1%) CIP/CIM. Diagnoses requiring ICU admission were widely and due to variable medical and surgical disorders. Two studies investigated the functional outcome in patients with ICUAW and coexistent brain lesions [45, 48]. Twenty (68.9%) studies were performed on subjects during their ICU or post-ICU stay, 1 in neurology and 8 (27.5%) in rehabilitation or in neuro-rehabilitative settings.

**Table 1:** Studies about functional outcome in CIPNM subjects. The pathological condition was counted as CIP if differentiation between types of CIPNM was not performed and if this acronym or definition was not specified in the studies analyzed

Authors	Study design/Setting/aim	N/followed/CIPNM type	Etiology	Follow-up	Functional measures/other	Other measures	Outcome
Zochodne DW et al^24^ (1987) [24]	case series, retrospective; single center; ICU; clinical and electrophysiological aspects	*N* = 19; (9 M, 10 F, mean age 64); CIP = 19	cardiac or pulmonary diseases; 5 pts had cerebral lesion (4 infarction, 1 brain injury)	10 mos-2 yrs	none	EMG, histological examination	8 (88.8%) pts showed good functional recovery. Of these 6 pts had EMG improvement within 3 months. At 2 yrs one patients had mildly weak dorsiflexion of right foot and one had mild distal limb weakness 11 (58%) pts died
Coronel B et al^25^ (1990) [25]	case series, retrospective; 2 ICU center; occurrence and clinical features	*N* = 15/4 (12 M, 3 F, mean age 47); CIP = 15	cardiac or pulmonary disorders	1–8 yrs	none	EMG, muscle biopsy	3 pts: 2 pts had persisting dysesthesia; 1 needing assistance to sit and walk; Death: 5 pts (33%)
Op de Cul et al^2^ (1991) [2]	case series, retrospective; ICU; clinical and electrophysiological features	*N* = 22^d^; (17 M, 5 F, mean age 55) CP = 22	Multiple trauma with brain injury (5 pts), pulmonary and infections	2–10 mos	none	EMG, muscle biopsy (7 pts)	9 (64.2%) pts had complete functional recovery; 5 (22.7%) pts: incomplete recovery; 8 pts died
Witt NJ et al^26^ (1991) [26]	case series, prospective; ICU; clinical and electrophysiological features	*N* = 30; CIP = 30, of these 25 had clinical signs of PN and 15 pts ES signs	multiple medical and surgical diseases; 25% had head trauma and brain lesions	mean 72 days (10–190)	none	EMG	20 (66.6%) pts gained full recovery; 3 (10%) with severe CIP showed severe disability and ultimately died 10 (33%) pts died
Rossiter A et al^27^ (1991) [27]	case reports, retrospective; single center; ICU; clinical report after pancuronium discontinuation	*N* = 5 pts; (4 M, 1 F); CIPNM = 5	medical disorders	5 mos	none	EMG; clinical examination	none had complete recovery: 1 pt severe disability at 3 months; 1 pt with tetraparesis was able to walk with assistance at 5 months; 1 pt with tetraparesis was unable to walk at 1 month; 2 pts died
Gooch JL et al^28^ (1993) [28]	case series, retrospective; paralysis after neuromuscular junction blockade	*N* = 12^b^; age range 3.5 mos-64 yrs; CIP = 12	medical disorders	3–6 mos	none	MRC; EMG, muscle biopsy (2 pts)	5 (50%) pts had recovery; 5 pts incomplete recovery; 2 (16.6%) pts died
Giostra E et al^29^ (1994) [29]	case series, retrospective; ICU; paralysis after neuromuscular junction blockade	*N* = 9; (6 M, 3 F, mean age 65.6 + 10.3); N = CIPNM	medical and pulmonary disorders	4 -52 wks	none	EMG, muscle biopsies (7 pts)	5 (55.5%) pts had complete recovery. Even if recovery was usual, residual peroneal palsy was frequent
Leijten F et al^30^ (1995) [30]	prospective cohort study; single center ICU, post-ICU; incidence and risk factors	*N* = 29^a^; CIP = 29 (21 M 8 F; mean age 59.7 ± 13.9 years); *N* = 12 evaluated to follow-up	surgical and medical disorders; 3 pts cerebral surgery, multiple trauma (*n* = 9), cardiac resuscitation (*n* = 5), intracranial hemorrhage (*n* = 2)	1 yr	none; endpoint was strength greater than MRC grade 4/5 in all muscles with ability to walk for more than 50 m without aid or ataxia	neurological examination; MRC; EMG;	7 (58.3%) patients recovered (4 pts within 3 days and 4 weeks, 3 pts within 4 weeks and 1 year; 5 (41.7%) pts had severe disability after year; 9 (31%) pts died
Latronico N et al^31^ (1996) [31]	case series, prospective; single center ICU; incidence and risk factors	*N* = 24; (19 M, 5 F, mean age 50.2 ± 20.9 yrs); CIPNM = 24	All patients had NCS lesions: 13 head trauma; 6 subarachnoid hemorrhage; 3 stroke; 1 cerebral hemorrhage	8–18 mos	none	EMG/ENG; nerve biopsy (22 pts)	7 survivors: 6 (85.7%) had recovered well or had only moderate disability (able to walk unassisted with full muscle strength); 1 was in vegetative state; 17 (70.8%) pts died
Berek K et al^32^ (1996) [32]	case series, prospective; ICU; incidence, severity and course of polyneuropathies in patients with sepsis or systemic inflammatory response syndrome	*N* = 22 with sepsis; (17 M, 5 F, mean age 51.2 yrs); CIPNM = 15	medical and surgical disorders	2–3 mos	functional disability score^$^	EMG	9 (50%) pts had complete functional recovery; 6 pts had incomplete functional recovery. Of these, 4 pts had mild weakness and 2 pts had moderate weakness Good tendency for recovery in all surviving patients, electrophysiologic findings were still pathologic in 11 patients at the follow-up; 7 (50%) pts died
Hund EF et al^33^ (1996) [33]	case series, prospective; single center ICU;	*N* = 7 (3 M, 4 F; mean age 47.7 ± 16.8	medical disorders; 3 pts with cerebral lesions	3 mos—3.5 yrs	none	EMG; muscle and nerve biopsy (3 pts)	2 (40%) pt gained complete recovery; 3 pts showed disability due to CNS lesions; 2 pts died
Campellone JV et al^34^ (1998) [34]	case series, prospective; single center ICU; frequency of myopathy as a cause of generalized weakness and potential risk factors after liver transplant	*N* = 7/6; (6 M, 1 F; mean age 57.7 ± 9.3) CIM = 7	liver transplant	11–41 days (5 pts) and 67 days (1 pt)	none	EMG; muscle biopsies (5 pts)	3 (50%) pts regained strength slowly and were able to ambulate within 4 to 12 weeks; 1 pt required a walker; 2 pts died
Lacomis D. et al^35^ (1997) [35]	cohort, retrospective; single center ICU; causes of ICU weakness	*N* = 92; *N* = 49 CIM = 37^e^ CIP = 12	surgical, medical and pulmonary disorders	12 – 60 mos	none	EMG; muscle biopsies (22 pts)	25 (75.7%) pts had complete functional recovery: 17 pts were ambulatory within 4 months and 8 pts within 4–12 months; 7 pts showed incomplete functional recovery: 4 remained non ambulatory and 3 remained dependent on the ventilator; 16 pts died
de Sèze M. et al^36^ (2000) [36]	cohort, retrospective; single center; rehabilitation; the features and outcome patients who had severe forms of CIP	*N* = 19, only CIP (14 M 5 F; mean age 55,9 yrs)	medical disorders	2 yrs	none	MRC; sensory findings	11 (64.7%) patients recovered completely; 4 (23.5%) patients remained quadriplegic; 2 patients remained quadriparetic; 2 pts died
Zifko UA et al^37^ (2000) [37]	cohort, retrospective; ICU and rehabilitation; clinical outcome and electrophysiological findings	*N* = 26; CIP = 13, (9 M, 4 F, age between 22–83 yrs); *N* = 7 refused to participate;	medical disorders; 1 pt with stroke	13–24 mos (mean 17 mos)	none	EMG/ENG; MRC; clinical examination	only 2 (15.3%) pts had full recovery; 11 of 13 patients with CIP had clinical manifestations, at follow-up (13–24 months after diagnosis); 6 pts died
16 De Jonghe B. et al^38^ (2002) [38]	cohort, prospective; multicenter ICU and post-ICU; clinical incidence, risk factors, and outcomes of ICU acquired paresis (ICUAP) during ICU stay	*N* = 95; CIP (ICUAP) = 24; (12 M, 12F; mean age 67,6 yrs)	surgical and medical disorders; patients were excluded if they had disease of the peripheral nervous system, or brainstem lesions	9 mos	none	MRC; EMG; muscle biopsy (10 pts)	15 (88.6%) patients had recovered an MRC score of 48 or higher at follow-up; 1 pt lost to follow-up; 7 pts died
Fletcher S.N. et al^39^ (2003) [39]	cohort, prospective study; multicenter post-ICU; prevalence, clinical characteristics and electrophysiological features	*N* = 22; CIP = 22; (mean age 62 yrs, range 45–78);	surgical and medical disorders	3.5 yrs (range, 12–57 mos)	Barthel Index	neurologic examination; EMG	19 (86.3%) pts had showed recovery quantified to BI between 95–100; 2 pts had recovery with BI score 85; 1 pts severe disabled. 95% patients had electromyographic evidence of chronic partial denervation, indicative of a preceding axonal neuropathy
Kerbaul et al^6^ (2004) [6]	cohort, prospective; single center post-ICU; to describe patterns of neuromuscular weakness by EMG and biopsy; functional outcome	*N* = 15 pts; (9 M, 6F; median age 53 yrs, range 33–82); CIP = 6 CIM = 6; CIP/CIM = 3	heart-surgery	12 mos	none, the endpoints were death or time to ambulation without assistance;	EMG; muscular/nerve biopsy (all pts)	6 (75%) had good recovery; 2 subjects of the 8 survivors were not ambulatory; 7 (46%) pts died
Van der schaaf M et al^40^ (2004) [40]	prospective observational cohort study and cross-sectional study^c^; single center ICU, post-ICU; to evaluate the functional outcome of ICU patients	*N* = 16; (12 M, 4 F; mean age 67 years); CIP = 16	medical and surgical disorders; patients with neurological disorders due CNS injury were excluded	6 mos and 1 yr	Barthel Index; Jebsen hand function test; rivermead mobility index; timed UP & GO walking test	MRC; SIP-68; SF-36; IPA questionnaire	At 6 mos, 8 pts were evaluated and all showed disability (activity and participation); median sumscore Barthel Index was 18.5 (range 9–20) and rivermead mobility index was 11 (range 1–14). At 1 year, 5 (31.2%) pts were evaluated. Improvement in functional abilities with wide variation in functional outcome among the patients, but functional impairment was still dominant in four out of 5 surviving pts. Outdoor mobility was reduced. All pts, excepts for one judged their quality of life as unsatisfactory in many areas 9 (56.2%) patients died
Guarneri B et al^22^ (2008) [22]	prospective cohort; multicenter post-ICU; to evaluate the long-term follow-up	*N* = 92; CIP = 15, (12 M 3 F; mean age 44.7 ± 14.9 yrs); CIP = 4 CIM = 6 CIP/CIM = 3 2 = undetermined	surgical and medical disorders; (intracerebral haemorrhage, metabolic encephalopathy, post-anoxic encephalopathy: 1 patient each); 5 multiple trauma patients; 3 head trauma	1 yr	global motor performance^$^	MRC; EMG; neurological examination	8 (61.5%) patients recovered; 2 (13.3%) patients had persisting muscle weakness; 1 patient remained tetraparetic; 1 patient remained tetraplegic; 1 patient lost to follow-up; 2 patients died;
Intiso D et al^41^ (2011) [41]	cohort prospective; single center neuro-rehabilitation; to evaluate the long-term functional outcome and health status	*N* = 42 (23 M, 19F, mean age 58.4 ± 13.9); CIP = 30 CIM = 6 CIP/CIM = 6	19 pts had CNS damage	5 yrs; mean 31.7 ± 15.8 months	Barthel and modified Rankin Scales (mRS);	SF-36 questionnaire	31 (73.8%) pts (24 pts with just CIPNM and 7 pts with CIPNM and CNS involvement) gained good recovery: mean Barthel of 86.7 ± 15.9 (*P* < 0.001), and the median mRS of 1 (IQR: 0–3), respectively, at follow-up (mean 31.7 ± 15.8 months)
Novak P et al^42^ (2011) [42]	cohort, prospective; single center rehabilitation; outcome to ICF	*N* = 27; (16 F, 11 M; mean age 59.4 ± 15.9); CIP = 27	not reported	from admission to discharge (9–102 days)	FIM; 6-min (expressed in meters) and 10-m walking test (expressed in speed velocity); ICF check list	sum of muscles strength;	Significant functional improvement; mean FIM score 78.7 ± 24.12 and 103.3 ± 20.5 at admission and discharge, respectively (*p* < 0.001); 6 -min walking test (m): 77.3 ± 115.3 and 191.5 ± 178.2, at admission and discharge, respectively (*p* < 0.001). Considering ICF, 26 (96.2%) pts improved activities and participation
Semmler A. et al^43^ (2013) [43]	cohort, retrospective observational; single center post-ICU; long-term outcome	*N* = 51; (26 M, 24 F; median age 57 yrs, range 19–75); CIP = 21, no CIM or CIP/CIM	Subjects with CNS lesion were excluded	6–24 mos, median 11 mos	ODSS^$^; median ODSS scores 1 (range 0–8);	MRC; median MRC sum scores 56 (range 47–60); EMG/ENG; neurological examination	Good recovery; pts with diagnosis of CIP showed a higher ODSS scores 1 (range 0–8) versus 0 (range 0–5); *p* < 0.001 and lower MRC sum scores 56 (range 47–60) versus 60 (range 58–60); *p* < 0.001. The neuromuscular long-term consequences of critical illness were not severe, suggesting a favorable prognosis of ICU-acquired muscular weakness
Koch S et al^23^ (2014) [23]	Prospective cohort; post-ICU; prediction of long-term outcome in CIP and CIM	*N* = 26; (20 M, 6 F; mean age 46 yrs); CIM = 8, CIP/CIM = 11, Control = 7	multiple trauma (*n* = 12)	1 yr; (mean 411 ± 121 days)	functional health status^$^	MRC; EMG; dmMCAP, neCMAP; neurological examination	4 (50%) of the CIM patients reached normal physical capacity. In contrast, only 3 (27%) of CIM/CIP patients did so at 1 year. Four (36%) of CIM/CIP pts still needed assistance to perform daily life activities: 2 pts were able to walk only within their homes and 2 were only able to stand with help or not at all. MRC sum scores assessed at follow-up examination were significantly lower in partially recovered patients (*n* = 6) compared with fully recovered patients (*n* = 20) [MRC sum score (median and 25th/75th percentiles): 48 (54/46) vs. 60 (60/57)]
Nguyen The N et al^44^ (2015) [44]	Cohort, prospective longitudinal observational; single center; neurology; incidence and distribution of CIP/CIM subtypes and the evaluation of the risk factors and outcomes	*N* = 133 pts *N* = 73 pts; CIP = 35; CIM = 16; CIP/CIM = 22; controls = 60	medical disorders	3 mos	none	MRC; EMG; ONLS	At the end of the follow-up duration (90 days), 31 pts with CIP/CIM were evaluated: the ONLS scores improved but remained significantly higher in comparison to non-CIP (2.7 vs 0.8, *p* = 0.015); 36 (49%) died
Intiso D et al^45^ (2017) [45]	prospective cohort study; single center, neuro-rehabilitation setting; functional recovery in subjects with sABI and CIPNM	*N* = 36; (27 M, 9 F, mean age 56.2 ± 14.8 yrs) CIP = 36; *N* = 75 controls (sABI)	patients with sABI	107 days (65–146)	LCF, DRS, GOS, mRS	LOS	The magnitude of these improvements was different between the groups, showing that patients with sABI only had a better improvement than those with CIPNM + sABI for mRS and DRS at discharge Subjects with sABI + CPNM showed 25.94 (23.33–28.86), 19.71 (17.42–22.31) to DRS and 2.76 (2.51–3.05) and 3.12 (2.84–3.42) to GOS, at admission and discharge, respectively
Cunningham CJB et al^46^ (2018) [46]	prospective observational; case–control; rehabilitation setting; prevalence of CIPNM in rehabilitative setting and impact of CIPNM on function	*N* = 23, (19 M, 4 F, mean age 43.6 ± 14.7); CIP = 16; CIM = 2; CIP/CIM = 5 controls = 10	medical disorders, 12 pts had SCI 2 pts stroke and one TBI	1 yr	FIM; FIM gain and FIM efficiency	EMG/ES; rehabilitation length of stay (RLOS), and discharge disposition	FIM score: 64.1 and 89.7 at admission; 78.4 and 94.6, at discharge in pts with CIPNM and without CIPNM, respectively. The gains in FIM scores and RLOS were greater, leading to similar FIM efficiency (FIM points gained/day of rehabilitation) compared with those without CIPNM (only for 13 pts). Those with CIPNM were less likely to be discharged directly home (57% versus 90%). At 1 year, recovery was seen in 80% of those with CIM and 55% of those with CIM/CIP
Symeonidou Z et al^47^ (2019) [47]	multicenter; retrospective observational; rehabilitation setting; functional recovery	*N* = 28 pts (19 M, 9 F, mean age 53.6 ± 14.5); CIP = 28	medical disorders; cerebral or spinal cord injury or stroke were excluded	109.4 ± 70.7 days	Barthel Index; ADL	MRC, sensory examination	Mean Barthel score at admission and discharge improved significantly (15.3 ± 9.1 vs 63.6 ± 21.6, *p* < 0.05); 3 (10.7%) pts had Barthel score > 85; 13 (46.4%) pts showed Barthel score 65–80; 5 pts had severe Barthel score 0–40, at discharge
Hakiki B et al^48^ (2021) [48]	Single; retrospective observational; rehabilitation setting; functional recovery	*N* = 224 pts; (81 (36%) females, age 68.73); CIPNM = 119	patients with sABI	3.8 mos	CRS-R; FIM; GOS-E; FOIS	ENG/EMG	All patients gained functional improvement at discharge for FOIS, FIM and GOS-E (*P* < 0.001). Those with a concomitant CIPNM achieved significantly lower scores for FIM (18.0 [1.0] and 20.0 [13.0] vs. 18.0 [6.0] and 37.0 [60.0] at the entry and discharge, respectively (*P* < 0.001); and GOS-E (3.0 [1.0] and 3.0 [1.0] vs. 3.0 [1.0] and 3.5 [2.0], at the entry and discharge, respectively (*P* < 0.001). The CIPNM absence was associated with a higher probability to achieve functional autonomy

*ADL* activity daily living, *DRS* disability rating scale, *GOS* Glasgow outcome scale, *dmCMAP* direct muscle stimulation, *ES* electrophysiological studies, *FIM* Functional independence measure, *ICF* International Classification of Functioning, Disability and Health, *ICUAP* Intensive Care Unit acquired paresis, *IPA* Impact on Participation and Autonomy questionnaire, *LCF* Levels of Cognitive Functioning, *LOS* length of stay, *MRC* Medical Research Council scale, *mRS* modified Rankin Scale, *ODSS* Overall Disability Sum score, *ONLS* Overall Neuropathy Limitations Scale, *neCMAP* nerve stimulation, *RLOS* rehabilitation length of stay, *RMI* Rivermead mobility index, *sABI* severe acquired brain injury, *SCI* spinal cord injury, *SIP-68* Sickness Impact Profile, *SF 36* Short Form 36 questionnaire, *TBI* traumatic brain injury, *CRS-R* Coma Recovery Scale-Revised, *GOS-E* Glasgow Outcome Scale-Expanded, *FOIS* Functional Oral Intake Scale

^a^number of patients who had polyneuropathy to EMG

^b^the sample included children and CIP was not defined

^C^two simultaneous studies on the one year-course: a prospective cohort study and a cross sectional study at same centre in different time period

^d^including 12 patients described in a precedent paper

^e^other forms of myopathy or motor axonopathy could not be excluded; $ description is reported in appendix 2

### Functional outcomes

Determining the functional outcome was the main purpose of 9 (31%) studies [22, 23, 40–43, 45, 47, 48]. Overall, 70.3% of subjects with ICUAW achieved a full recovery. Eighteen studies reported a percentage above 50% and among these, 10 showed that 75% of the sample (range 75–100) reached a full recovery. Functional measures were variable and overall, 16 tools were used to evaluate recovery: the Barthel Index (39–41, 47), the Functional Independence Measure (FIM) [42, 46, 48], the modified Rankin scale (mRS) [41, 45], the activity daily living scale (ADL) [47], the Disability Rating scale (DRS) [45], the Glasgow outcome scale (GOS) [45], Glasgow Outcome Scale Extended (GOS-E) [48]; the Coma Recovery Scale- Revised (CRS-R) [48]; the Rivermead Motor Assessment Scale (RMA) (40), the functional disability, the Jebsen hand function test [40], the timed UP & GO walking test [40], the global motor performance [22], the 6-min (expressed in meters) and 10-m walking test (expressed in speed velocity) [42], the Overall Disability Sum score (ODSS) [43], and the Functional Health Status [23]. The Barthel Scale and the FIM were employed in 4 [39–41, 47] and 3 studies [42, 46, 48], respectively. The studies that used the Barthel scale showed contrasting findings. Of these, 2 prospective studies reported that 86.3% and 73.8% of the sample, respectively, achieved a good recovery (mean Barthel score > 90) at follow-up [39, 41]. Conversely, 10.7% and 31.2% of subjects made a full recovery in the remaining 2 studies [40, 47], respectively. The studies that used the FIM showed a significant functional improvement. In this regard, Novak et al. reported that subjects with CIP had a mean FIM score of 78.7 ± 24.12 and 103.3 ± 20.5, at admission and discharge, respectively (*p* < 0.001) [42]. Likewise, a prospective observational case control study by Cunningham et al. found that subjects with ICUAW had greater gains in FIM scores, leading to similar FIM efficiency (FIM points gained/day of rehabilitation), than subjects without ICUAW [46]. However, despite these seemingly functional improvements and similar discharge FIM scores, subjects with ICUAW were less likely to be discharged directly home (57% versus 90%).

Fourteen (48.2%) studies did not have patient’s global ability as primary end-point and did not use functional scales. Recovery was evaluated from a clinical point of view by neurological examination or on the basis of the improvement in muscle strength by the MCR scale. This measure was used in 11 studies [22, 23, 28, 30, 36, 38, 40, 42–44, 47]. The severity of muscle weakness was not correlated with the clinical and electrophysiological diagnosis, and there was no correlation between the degree of the nerve conduction and the clinical findings [22, 37].

### Long-term outcomes

Seven studies (24.1%) had a follow-up longer than 1 year, ranging from 2 to 8 years (Table 2) for a total of 173 (21.9%) subjects, of whom 37 (21.3%) died and 108 were followed. Overall 124, 43 and 6 subjects had CIP, CIM and CIP/CIM, respectively. Of followed subjects with ICUAW 96 (88.8%) achieved a full recovery. Almost all studies included small samples characterized by case series including 7 to 22 patients, and only 2 studies had greater samples consisting of 49 [35] and 42 subjects [41], respectively. Furthermore, the investigation of long-term functional outcomes in subjects with ICUAW as main purpose was addressed only in 1 of these 7 studies [41]. This study had a mixed sample and included 42 patients with different etiology of ICU admission and showed that 73.8% of the entire sample of patients made a good recovery, as shown by the functional measures at follow-up: mean Barthel and median mRS score of 86.7 ± 15.9 and 1 (IQR: 0–5), respectively. The differentiation between the types of CIPNM was performed only in 2 studies [35, 41]. Both studies showed that subjects with CIP/CIM had the worst outcome. Functional measures were employed only in 2 studies that used the Barthel scale [39, 41] and the mRS [41]. Both studies had a long follow-up of 3.5 and 5 years, respectively, and a good recovery was detected in a high percentage of subjects. Five (71.4%) studies did not use proper scales, and the recovery was evaluated by motor improvement, such as the ability to walk without support or aid.

**Table 2:** Long-term functional outcome in subjects with critical illness polyneuropathy and myopathy

Authors	N/followed/CIPNM type	Follow-up	Functional measures	Other measures	Outcome
Zochodne DW et al^24^ (1987) [24]	*N* = 19; (9 M, 10 F, mean age 64); CIP = 19	10 mos-2 yrs	none	EMG, histological examination	8 (41.1%) pts showed good functional recovery; of these 6 pts had EMG improvement within 3 months. One pt had mild distal limb weakness at 12 wks and one had mildly weak dorsiflexion of right foot, at 2yrs; 11 (58%) pts died
Coronel B et al^25^ (1990) [25]	*N* = 15/4 (12 M, 3 F, mean age 47); CIP = 15	4–8 yrs	none	EMG, muscle biopsies	3 pts: 2 pts had persisting dysesthesia; one needing assistance to sit and walk death: 5 pts (33%)
Hund EF et al^33^ (1996) [33]	*N* = 7 (3 M, 4 F)	3 mos to 3.5 yrs	none	EMG; muscle and nerve biopsy (3 pts)	2 (28.5%) pt gained complete recovery; 3 (42.8%) pts showed disability due to CNS lesions; 2 pts died
Lacomis D. et al^35^ (1997) [35]	*N* = 92; N = 49 CIM = 37^a^; CIP = 12	12 – 60 mos	none	EMG; muscle biopsies (22 pts)	25 (51%) pts had complete functional recovery: 17 pts were ambulatory within 4 months and 8 pts within 4–12 months; 7 pts showed incomplete functional recovery: 4 pts remained non ambulatory and 3 remained dependent on the ventilator; 16 pts died
de Sèze M. et al^36^ (2000) [36]	*N* = 19, only CIP (14 M 5 F; mean age 55,9 yrs)	2 yrs	None	MRC; sensory findings	11 (57.8%) patients recovered completely; 4 (21%) patients remained quadriplegic; 2 patients remained quadriparetic; 2 pts died
Fletcher S.N. et al^39^ (2003) [39]	*N* = 22; CIP = 22; 62 yrs (45–78);	3.5 yrs (range, 12–57 mos)	Barthel Index	neurologic examination; EMG	19 (86.3%) pts had recovery quantified to BI 95–100; 2 pts incomplete recovery (BI score 85); 1 pts severe disabled; 95% patients had EMG evidence of chronic partial denervation
Intiso D et al^41^ (2011) [41]	*N* = 42 (23 M, 19F, mean age 58.4 ± 13.9); CIP = 30 pts; CIM = 6 pts CIP/CIM = 6 pts	5 yrs; mean 31.7 ± 15.8 months	Barthel scale and mRS	SF-36 questionnaire	31 (73.8%) pts (24 pts with just CIPNM and 7 pts with CIPNM and CNS involvement) gained recovery with a mean Barthel of 86.7 ± 15.9 (*P* < 0.001), and the median mRS of 1 (IQR: 0–3), respectively, at follow-up (mean 31.7 ± 15.8 months)

*ES* electrophysiological studies, *MRC* Medical Research Council scale, *mRS* modified Rankin Scale, *ODSS neCMAP* nerve stimulation; Overall Disability Sum score, *ONLS* Overall Neuropathy Limitations Scale, *LOS* length of stay, *SF 36* Short Form 36 questionnaire, *TBI* traumatic brain injury

^a^other forms of myopathy or motor axonopathy could not be excluded

### ICUAW type and outcome

The differentiation between the types of ICUAW was performed in 7 (25%) studies [6, 22, 23, 35, 41, 44, 46]. Among these, 3 investigations concerning the long-term functional outcome reported that the CIM type had a better prognosis and an earlier recovery than CIP/CIM [22, 23, 41]. In detail, Koch et al. enrolled a cohort of 26 subjects consisting of 11 and 8 patients with CIP/CIM and CIM, respectively, whereas the remaining 7 were controls. After 1 year of follow up, in 7 (87.5%) and 6 (54.5%) patients with CIM and CIP/CIM, respectively, a return to normal physical capacity and a normal EMG were observed. Furthermore, 50% of CIM patients recovered within 3 months, returning to a normal or at least a sufficient physical capacity to resume daily life activities. On the other hand, 5 (45.5%) patients with CIP/CIM had a partial recovery and abnormal electrophysiological findings. Guarneri et al. reported the long-term recovery of 15 patients with ICUAW and, of these, 4, 6 and 3 patients had CIP, CIM and CIP/CIM, respectively, whereas 2 subjects were undetermined. Five subjects with CIM recovered within 6 months, whereas the presence of CIP alone or in addition to CIM was associated with a more delayed recovery between 6 and 12 months, and more than 50% of those individuals had persistent deficits at 1 year of follow-up [22]. Likewise, the study by Intiso et al. reported that CIM patients had a better recovery than subjects with CIP or CIP/CIM and did not show differences in their health status compared to the Italian normative data. Of the remaining 4 studies, 3 did not report data on the recovery of ICUAW types, since ICUAW subjects were evaluated in comparison to controls independently of the type of ICUAW [6, 44, 46], and the last one had severe limitations despite the differentiation between the types of ICUAW, since other forms of myopathy or motor axonopathy could not be excluded [35].

## Discussion

The present review detected that 70.3% of a large number subjects with ICUAW could achieve a good recovery. This finding is similar to that obtained from a previous review by Latronico et al., who reported that 68.8% of patients made a complete recovery [21]. Furthermore, a higher percentage of 88.8% gained good recovery at long term follow-up. However, because of strong limitations of studies that had small samples and were widely variable in aim, methodology design, case mix, and outcome measures, the finding should be considered with caution. The investigation of the functional outcome was the main purpose in 31% of the studies analyzed; in addition, a variety of measures was employed, and only 7 (24.1%) studies used a proper functional scale such as the Barthel scale and the FIM.

Of great importance, immediately after the rehabilitation treatment, is the overall health status of the patients, culminating in a return to active daily living, socialization and participation. The International Classification of Functioning, Disability and Health (ICF) recommends a new approach to evaluate disabled people, which is based on a holistic model in which activities and participation represent essential aspects. In this respect, only 2 studies investigated the outcome according to the ICF, but because of the small size of the samples and the limited duration of the follow-ups, it was not possible to draw definitive conclusions [40, 42]. Most studies (71.4%) evaluated recovery on the basis of the motor improvement and the ability to walk without support, but no data was reported on the functionality in activity daily living. Furthermore, though the present review retrieved a larger number of 788 patients with ICUAW, those who had long term follow-up for more than 1 year were only 173 (21.9%) subjects.

Over two decades, until year 2000, the main purpose of investigators was to characterize the new clinical phenomenon. There were no studies having patient’s global ability and quality of life as primary end-points. The majority of these studies reported the outcome in terms of complete or incomplete recovery (30–31, 35–36), motor performance [30, 31] and ambulatory activity [6, 30, 35]. The outcome was also addressed, but it was generically labeled as “full recovery” or based on the achievement of the motor ability, particularly of ambulation without support or aid. A proper functional scale quantifying disability and evaluating the patients’ health status and quality of life was not employed. The evaluation of the functional outcome was performed on the neurological examination or on the improvement of the muscle strength by the MCR scale. Clinical recovery was considered complete if patients had an MRC grade of 4/5 in all muscles (22, 30). Although this scale has been recently demonstrated to be an important predictor of death and of a worse five-year survival [56], it was designed and validated to quantify muscle strength impairment but not in relation to functional abilities. Most studies were performed by ICU specialists, and this may explain the methodological approach used. In this respect, ICU specialists might prefer to assess muscle weakness and to diagnose early ICUAW during the ICU stay, since this disorder could be a severe complication for the weaning of mechanical ventilation [57, 58] and could delay ICU discharge.

Some authors suggested that patients with the Central Nervous System (CNS) injury might likewise develop critical illness polyneuropathy and, consequently, recovery might be hampered by the CNS damage (33, 35); however, only 4 (14.2%) studies [38, 40, 43, 47] excluded subjects with the CNS damage to avoid confounding findings on the outcome. On the other hand, 2 studies were designed to investigate recovery in subjects with severe acquired brain injuries (sABI) and coexistent ICUAW [45, 48]. The authors reported that patients with sABI and ICUAW achieved a good recovery, but the magnitude of these improvements was better in the subjects with sABI alone. However, it is still not clear if the residual disability in these subjects is due predominantly to ICUAW, to CNS damage or to both disorders. Likewise, the effect that each disease might have on the course of the disability still needs to be clarified. People with both disorders might require different rehabilitation approaches and strategies.

It has been suggested that subjects with CIM have a better prognosis, reaching early and full recovery, than subjects with CIP or CIP/CIM, but this finding was reported only in 3 studies including a total of 20 patients [22, 23, 41]. Therefore, it is not possible to achieve a definitive conclusion about this matter due to the very small number of investigated subjects. Several difficulties hamper and make it hard to make real and objective comments on this issue. Some of these difficulties are the lack of unique and shared definitions of muscle weakness that may affect ICU subjects as well as an insufficient differentiation between the types of ICUAW. Today, a wide range of definitions are still being used including ICUAW, CIP, CIM, CIP/CIM or CIPNM, and this aspect can complicate the analysis, and the results of the studies may be consequently biased. Most investigations considered this disorder as a single entity, therefore it is not possible to exclude that different forms of ICUAW were present in the sample of the studies investigating only subjects with CIP. Furthermore, although EMG is able to differentiate between the subtypes of ICUAW, it does not allow the quantification of the muscle impairment and the related disabilities, and no electrophysiological exams or imaging have helped to solve this point.

## Limitations

The present study has limitations that must be acknowledged. This is a review of the literature having the aim to discuss the recovery and the long-term functional outcome of ICUAW subjects. Cohort studies, case series as well as functional measurements and follow-up were highly heterogeneous regarding the functional outcome. A further limitation concerns the recovery of subjects with ICUAW, such limitation is due to the paucity of trials focusing on rehabilitative interventions [41, 42, 59]. Even if this issue is beyond the scope of the present review, the majority of the studies analyzed did not define whether the subjects followed any kind of rehabilitation treatment after hospital discharge. Currently, apart from the early neuromuscular electrical stimulation that might prevent ICUAW and improve the quality of life by enhancing muscle strength in ICU patients [59], no definitive studies have evaluated the effects of rehabilitation programs in inpatient or outpatient settings in this population [60]. Therefore, several questions remain unanswered and further research should be carried out on this matter.

## Suggestions and implications for the future

Given the protean aspects of ICUAW, a closer collaboration as well as a more active participation of multiple specialists and experts has been suggested [61]. In particular, ICU specialists, neurologists and physiatrists should collaborate more to properly evaluate and follow these subjects. Specialists who manage ICUAW patients should adopt unique and shared terminology and definitions, and future studies should be planned considering the following aspects:

1. the aim should be focused on functional recovery; the methodology design should include a large sample of patients, proper functional measures and defined long-term follow-up;
2. differentiation between the types of ICUAW;
3. rehabilitation interventions and their effect on functional outcome and quality of life, given that few studies have evaluated the effects of rehabilitation programs in this population.;
4. Occurrence and recovery of ICUAW in subjects with CNS damage should be investigated through dedicated studies.

## Conclusion

A percentage of 70.3% of survivor subjects with ICUAW could achieve a good recovery and a higher percentage was detected at long term follow-up. However, the quality of the published studies due to short follow-ups, and the absence of clearly defined outcome measures did not allow definitive conclusions. A close collaboration between specialists and proper planned research in this field are needed to answer the unsolved questions.

## Supplementary Information

**Additional file 1:** Appendix 1

**Additional file 2:** Appendix 2

## Data Availability

All data generated or analysed during this study are included in this published article [and its supplementary information files]. The datasets used and/or analysed during the current study available from the corresponding author on reasonable request.

## Abbreviations

ADL: Activity of daily living
DRS: Disability rating scale
GOS: Glasgow outcome scale
dmCMAP: Direct muscle stimulation
ES: Electrophysiological studies
FIM: Functional independence measure
ICF: International Classification of Functioning, Disability and Health
ICUAP: Intensive Care Unit acquired paresis
IPA: Impact on Participation and Autonomy questionnaire
LCF: Levels of Cognitive Functioning
LOS: Length of stay
MRC: Medical Research Council scale
mRS: Modified Rankin Scale
ODSS: Overall Disability Sum score
ONLS: Overall Neuropathy Limitations Scale
neCMAP: Nerve stimulation
RLOS: Rehabilitation length of stay
RMI: Rivermead mobility index
sABI: Severe acquired brain injury
SCI: Spinal cord injury
SIP-68: Sickness Impact Profile
SF 36: Short Form 36 questionnaire
TBI: Traumatic brain injury
CRS-R: Coma Recovery Scale-Revised
GOS-E: Glasgow Outcome Scale-Expanded
FOIS: Functional Oral Intake Scale
