# Supplementary material for: Recovery and long term functional outcome in people with critical illness polyneuropathy and myopathy: a scoping review
Source: BMC Neurol. 2022 Feb 11;22:50. doi: 10.1186/s12883-022-02570-z (PMC8831873; doi:10.1186/s12883-022-02570-z)
Supplement: Supplementary file 1 — Additional file 1: Appendix 1 [file 12883_2022_2570_MOESM1_ESM.docx]

**Appendix 1**

Excluded studies that enrolled subjects with ICUAW.

| **Authors** | **Study/**  **Design/**  **setting** | **N/followed/**  **CIPNM type** | **Etiology** | **Follow-up** | **Functional measures/**  **other** | **Other measures** | **Outcome** |
| --- | --- | --- | --- | --- | --- | --- | --- |
| Op de Cul A et al^48^ (1985) | case series;  ICU | N= 12/9;  CIP= 4;  CIP/CIM = 3 | eight pts had multiple trauma. Of these, 5 pts with brain concussion | 5 wks-5 mos | none | EMG, histological examination | 7(58%) pts gained complete functional recovery, 2 (16.6%) had incomplete recovery and 3 pts died |
| Gooch JL et al^49^ (1991) | case series;  retrospective | N = 12;  (mean age 34.2±20.5 yrs) | medical and pulmonary disorders | 3-6 mos | none | EMG, muscle biopsy (2 pts) | 8 (66.6%) pts had complete functional recovery within 6 months; 2 (16.6%) pts incomplete functional recovery and 2pts pts died (17%) |
| Lacomis D et al^50^ (1996) | case series;  retrospective; ICU | N= 14/10;  9 M, 5 F;  CIM = 10 | medical disorders and organ transplant, in particular liver transplant | 1 wk- 12 mos | none | clinical, EMG/ENG,  histologic examination (14 pts) | 5 pts complete functional recovery;  5 pts incomplete functional recovery: 3 pts were walking with assistance at 6 weeks, 3 and 4 months, respectively;  1 pt had moderate weakness at 2 months, 1 pt was ventilator-dependent at 2 months;  2 pts died |
| Koch S et al^51^ (2011) | single center;  cohort;  prospective observational;  ICU | N= 53;  CIP= 1;  CIM= 16;  CIP/CIM= 20;  Unspecified= 3 | severe head trauma were excluded |  | none | MRC; EMG; dmMCAP, neCMAP | At discharge from ICU, 25% of patients with isolated CIM showed electrophysiological signs of recovery and significantly lower degrees of weakness. Recovery could not be observed in patients with combined CIM/CIP |
| Schmidt B and Rollnik D^52^ (2016) | cohort;  single center; retrospective;  rehabilitation | N= 159 (90 M, 69 F, mean age 66±11)  CIP= 103 clinically diagnosed;  CIP= 56 diagnosed by ES exam | N= 52 pts had diabetes mellitus | not reported | Barthel Index; (value score not reported) | neurography; LOS | Outcome and prognosis of patients with confirmed CIP was not different from patients without neuropathy. |
| Kelmenson DA et al^53^ (2018) | cohort with propensity score matched analysis; retrospective | N= 3.567 pts  with a discharge diagnosis of CIPNM by ICD-9 code during 2010-2014 | medical and pulmonary disorders | not reported | not reported |  | Patients with a discharge diagnosis of CIPNM had fewer 28-day hospital free days (6 [0.1] vs 7.4 [0.1] days, p<0.0001), fewer 28-day ventilator free days (15.7 [0.2] vs 17.5 [0.2] days, p<0.0001), were less likely to be discharged home (15.3% vs 32.8%, p<0.0001) |
| Meyer-Frießem CH et al^54^ (2020) | cohort study; single center | N = 149 ICU survivors;  (mean age 59.5 ±13.4 yrs)  N= 95 pts with  ICUAW diagnosis according the German International  Classification of Diseases-10 (ICD-10) 2007-2017;  N = 33 pts without ICUAW | heart disease and trauma or burn injuries | ICU discharge-10 yrs | not reported | phone interview | Of all surveyed ICU survivors, 40% reported persistent symptoms at the time of the interview. 62% had persistent symptoms up to 10y after ICU (5-10y: 46%). Only 37% of participants reported a complete recovery of symptoms, significantly associated with an initially low number of symptoms after ICU (p < 0.0001). 73.7% of individuals reported impaired QoL, with 29.8% describing the impairment as “severe” and 65% (n =37/57) complaining about limitations in terms of daily life and leisure time |

Legend: dmCMAP= direct muscle stimulation; ES= electrophysiological studies; ICD-9= International Classification of Diseases -9; ICU= Intensive Care Unit; MRC = Medical Research Council scale; LOS= length of stay; neCMAP= nerve stimulation.
