# Supplementary material for: Recovery and long term functional outcome in people with critical illness polyneuropathy and myopathy: a scoping review
Source: BMC Neurol. 2022 Feb 11;22:50. doi: 10.1186/s12883-022-02570-z (PMC8831873; doi:10.1186/s12883-022-02570-z)
Supplement: Supplementary file 2 — Additional file 2: Appendix 2 [file 12883_2022_2570_MOESM2_ESM.docx]

**Appendix 2**

**Functional disability score** (Berek K et al 1996)

Graded from 0 normal neurological examination and functional state to 4: severe limitation of function without self-ambulation.

0 =Normal neurologic examination and normal functional state; 1 = Areflexia, with or without subjective symptoms and normal functional state; 2 = Neurologic findings other than areflexia, without or with only mild limitation of normal function; 3 = Neurologic findings other than areflexia with moderate or severe limitation of function; 4 = Severe limitation of function without self-ambulation (tetraplegia).

**Global motor performance** (Guarneri et al 2008)

It consisted of standing up from a chair, and walking unaided for at least 50 m with no evidence of ataxia (Leijten FS, Harinck-de Weerd JE, Poortvliet DC, et al. The role of polyneuropathy in motor convalescence after prolonged mechanical ventilation. JAMA 1995;274:1221–5).

**Overall Disability Sum score (ODSS)** (Semmler A et al. 2013)

ODSS is composed of an arm and leg disability scale with a total score ranging from 0 (no signs) to 12 (severe disability). The ODSS gives a description of arm and leg function in daily activities in a checklist form, such as dressing, doing and undoing buttons, or walking.

**Functional Health Status** (Koch S et al. 2014)

Standardized questionnaire with scoring as follows: 0= not able to stand at all; 1= stand with support; 2= able to walk within the room/flat alone, need some help with body care; 3= short distance walk (up to 500 m) is possible, need help for daily activities (housekeeping); 4= daily life needs and profession can be managed without help, but there are still limitations in activities (sport, professional, and private activities); and 5= full recovery, no limitation in daily life or in professional and private activities.

**Overall Neuropathy Limitations Scale** (ONLS) (Nguyen The N et al 2015)

The ONLS is scored from 0 to 5 on the upper limb section and from 0 to 7 on the lower limb section. The ONLS score ranges from 0 to 12, with 0 as normal and 12 as maximum disability (unable to make any purposeful movement of both arms and legs).
